# Supplementary material for: On the Origins of Omicron’s Unique Spike Gene Insertion
Source: Vaccines (Basel). 2022 Sep 9;10(9):1509. doi: 10.3390/vaccines10091509 (PMC9504260; doi:10.3390/vaccines10091509)
Supplement: Supplementary file 1 [file vaccines-10-01509-s001.zip › vaccines-1796862-supplementary-conversion.pdf]

Supplemental Material

Index:

<u>Figure S1</u>: Mutational burden of all SARS-CoV-2 proteins for the Omicron variant (B.1.1.529/BA.1/BA.2) compared with the Delta variant (B.1.617.2).

<u>Figure S2</u>: Possible nucleotide insertions giving rise to ins214EPE in the Omicron genome.

<u>Figure S3</u>: Alignment and normalized homology scores for regions flanking insertion and origin sequences for previously identified template switch-mediated insertions in the SARS-CoV-2 genome.

<u>Figure S4</u>: Distributions of normalized homology scores for regions flanking ins214EPE and candidate origin sequences.

<u>Figure S5</u>: Alignment of the Omicron genomic region corresponding to ins214EPE with the human TMEM245 transcript.

<u>Figure S6</u>: Occurrence of deletions and substitutions in the residues neighboring position 214, in SARS-CoV-2 genomes harboring any insertion at this position.

<u>Table S1</u>: Comparison of the mutations between the Omicron variant and previously identified variants of concern (VOCs) and variants of interest (VOIs).

<u>Table S2</u>: Number of PANGO lineages with mutations in the Omicron variant's Spike protein.

<u>Table S3</u>: List of other insertions near amino acid position 214 of the SARS-CoV-2 Spike glycoprotein.

<u>Table S4</u>: Prevalence of ins214EPE in Omicron sublineages.

<u>Table S5</u>: SARS-CoV-2 insertions identified and attributed to template switching in the previous analysis by Garushyants, et al.

<u>Table S6</u>: Sequences in the Omicron genome with a single nucleotide mismatch compared to the three candidate insertion sequences.

Table S7: Number of genomes with exact matches to candidate insertions by Pango lineage.

Table S8: Number of seasonal or enteric *Coronaviridae* genomes with exact matches to each candidate insertion sequence.

Table S9: Coexpression analysis of ACE2 and ANPEP in single cell RNA-sequencing datasets.

Table S10: Coexpression analysis of ACE2 and DPP4 in single cell RNA-sequencing datasets.

Table S11: Local alignment of 35 nucleotides upstream or downstream of origin and insertion sites from previously identified template switch-mediated insertions.

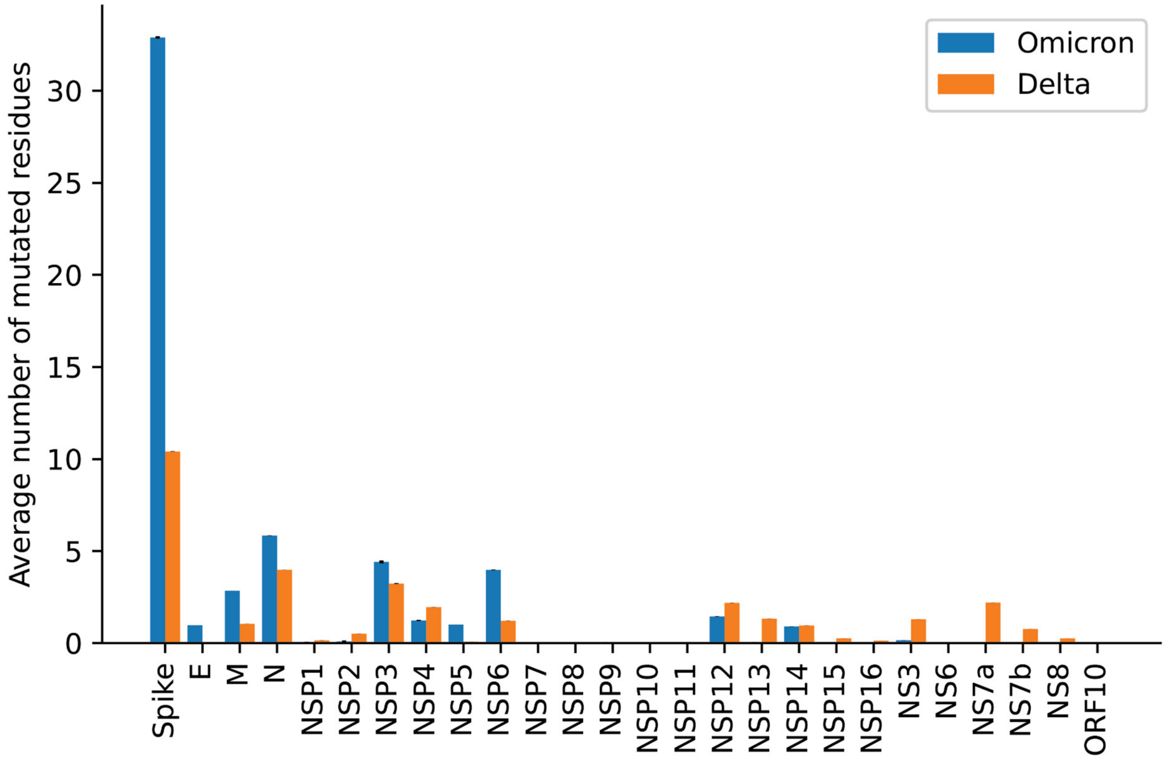

Figure S1. Mutational burden of all SARS-CoV-2 proteins for the Omicron variant (B.1.1.529/BA.1/BA.2) compared with the Delta variant (B.1.617.2). A total of 5,441 Omicron (B.1.1.529/BA.1/BA.2) sequences and 159,981 Delta (B.1.617.2) sequences were retrieved from the GISAID on December 16, 2021. Each bar represents the average number of mutations reported for a sequence in each of the SARS-CoV-2 proteins. Error bars represent the standard error of the mean.

```
                                                         22205  22206
Reference Sequence:        22190   5'-auu aau uua gug cgu gau cuc ccu cag ggu uuu-3'22231
                             210       I   N   L   V   R   D   L   P   Q   G   F    220

9-nt Insertion after ga:           5'-auu a-- -ua gug cgu gaG CCA GAA GAu cuc ccu cag ggu uuu-3'
                                       I     I     V   R   E   P   E   D   L   P   Q   G   F

9-nt Insertion between ga:         5'-auu a-- -ua gug cgu gAG CCA GAA Gau cuc ccu cag ggu uuu-3'
                                       I     I     V   R   E   P   E   D   L   P   Q   G   F

9-nt Insertion before ga:          5'-auu a-- -ua gug cgu GAG CCA GAA gau cuc ccu cag ggu uuu-3'
                                       I     I     V   R   E   P   E   D   L   P   Q   G   F
```

Figure S2. Possible nucleotide insertions giving rise to ins214EPE in the Omicron genome. There are three possible scenarios in which a 9-nucleotide insertion could yield the observed sequence. Because of the local sequence alignment (i.e. the occurrence of the insertion at a 5′-GA-3′ and the presence of a 5′-GA-3′ on either side of the insertion), it cannot be determined which scenario actually gave rise to the insertion.

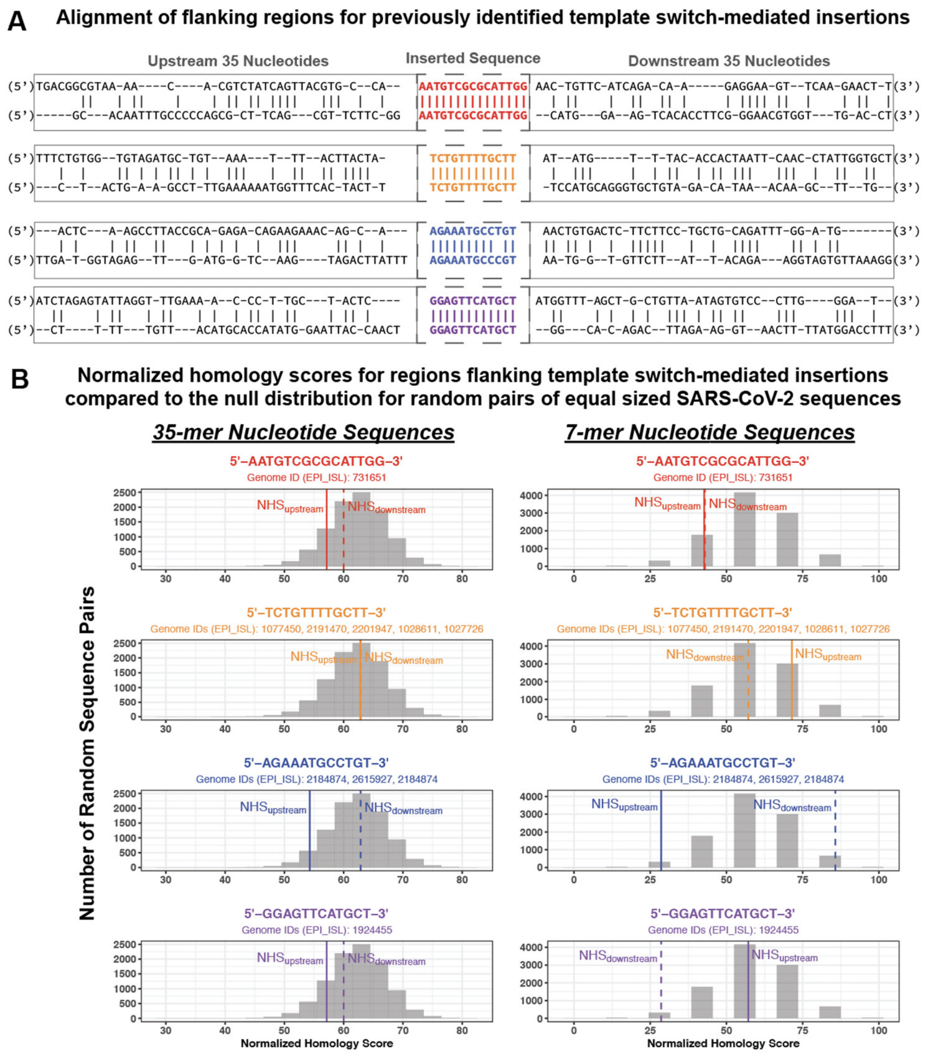

Figure S3. Alignment and normalized homology scores for regions flanking insertion and origin sequences for previously identified template switch-mediated insertions in the SARS-CoV-2 genome. Four "positive control" template switch-mediated insertions of 12 to 15 nucleotides were identified from the previous analysis by Garushyants et al.[14] We identified the corresponding SARS-CoV-2 genomes in GISAID for each insertion. (A) Alignment of the insertion (top) and origin (bottom) sites for the 35 nucleotides upstream and downstream of each inserted sequence. These alignments are also shown in **Table S8** for each individual genome. (B) For a given insertion, we then calculated normalized homology scores (NHS) between the regions flanking the inserted sequence and the origin (template) sequence. Specifically, we compared the 35 nucleotides upstream of the insertion to the 35 nucleotides upstream of the origin ("$NHS_{upstream}$"), and we separately compared the 35 nucleotides downstream of the insertion to the 35 nucleotides downstream of the origin ("$NHS_{downstream}$") (panel on left). We did the same to compare 7-nucleotide sequences upstream and downstream of the insertion and origin (panel on right). In each case, the normalized homology scores are shown for each insertion as vertical lines (solid line for upstream, and dashed line for downstream) on top of a control null distribution (gray histogram). The numerical scores are given in **Table S8**. To generate the null distributions of NHS values, we compared 10,000 pairs of non-

overlapping 35-nucleotide (or 7-nucleotide) sequences from the original SARS-CoV-2 genome (NC_045512.2).[79]

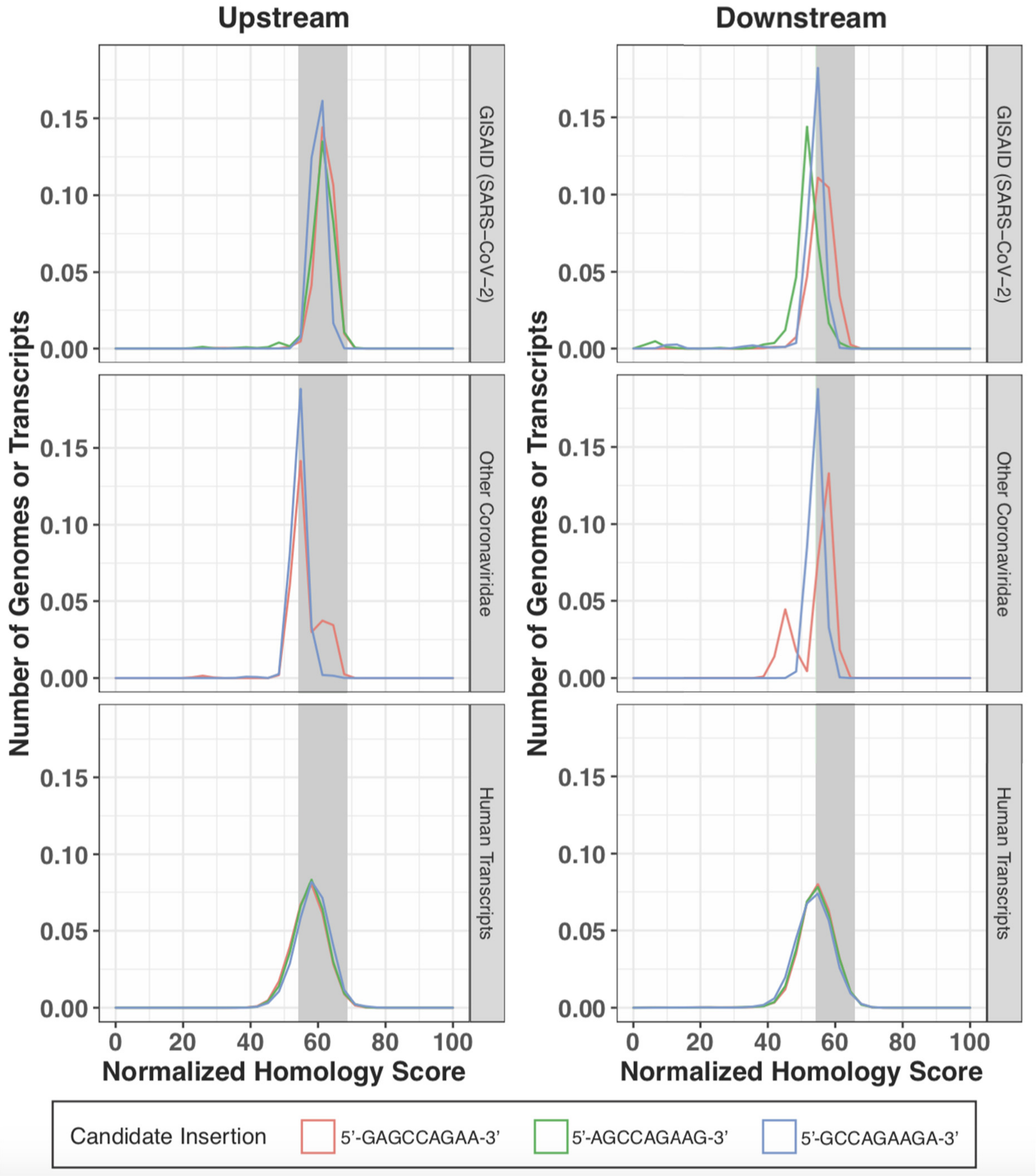

Figure S4. Distributions of normalized homology scores for regions flanking ins214EPE and candidate origin sequences. Distributions of normalized homology score (NHS) values between the regions flanking the Omicron insertion and the regions flanking the candidate origin sequences from SARS-CoV-2 genomes in GISAID (top), non-SARS-CoV-2 *Coronaviridae* genomes (middle), and human transcripts (bottom). Distributions are shown separately for the comparisons of nucleotide sequences upstream and downstream

of the Omicron insertion and the respective putative templates. The gray box in each plot corresponds to the 5th to 95th percentile of the NHS distribution comparing randomly selected non-overlapping 35-nucleotide sequences from the SARS-CoV-2 genome (i.e. the histogram shown in each panel of Figure S3.

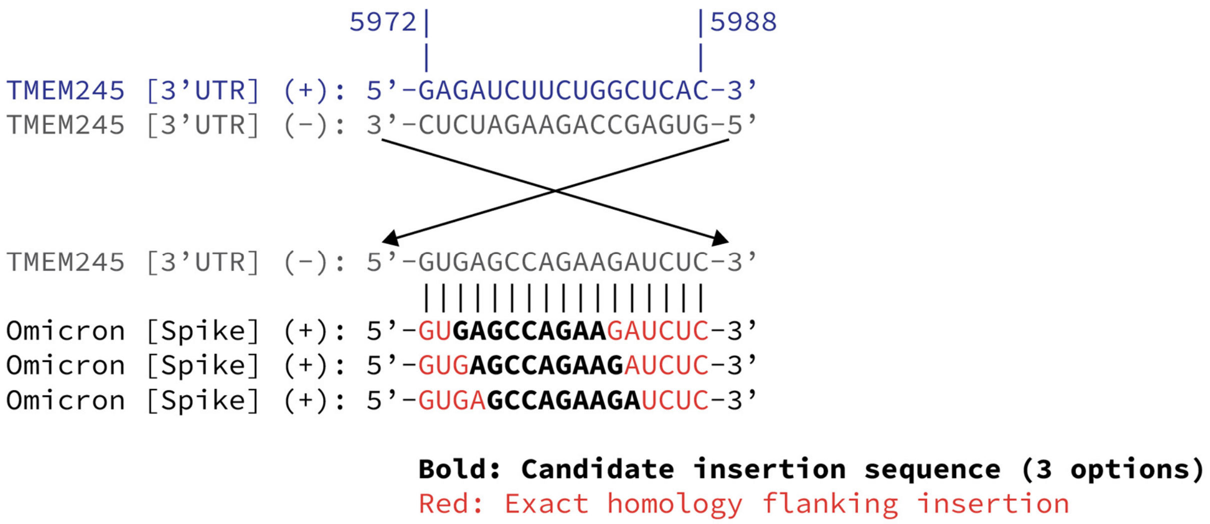

Figure S5. Alignment of the Omicron genomic region corresponding to ins214EPE with the human TMEM245 transcript. TMEM245 is an example of a human transcript which harbors the sequence that corresponds to ins214EPE and has exact homology in the short nucleotide sequences upstream and downstream of it. The relevant region of the TMEM245 transcript (nucleotides 5972 to 5988), which is present in the 3′ untranslated region (3′ UTR), is shown in blue. The reverse complement of this sequence is shown in gray, first in the 3′ to 5′ orientation and then flipped to visualize the 5′ to 3′ orientation from left to right. This sequence aligns exactly with the region corresponding to and surrounding ins214EPE in the Omicron genome. The three rows corresponding to the Omicron genome show identical sequences, but consider the three different candidate insertions that may have given rise to ins214EPE (see Figure S2). In each row, the candidate insertion sequence is shown in bold, and the flanking nucleotides are shown in red.

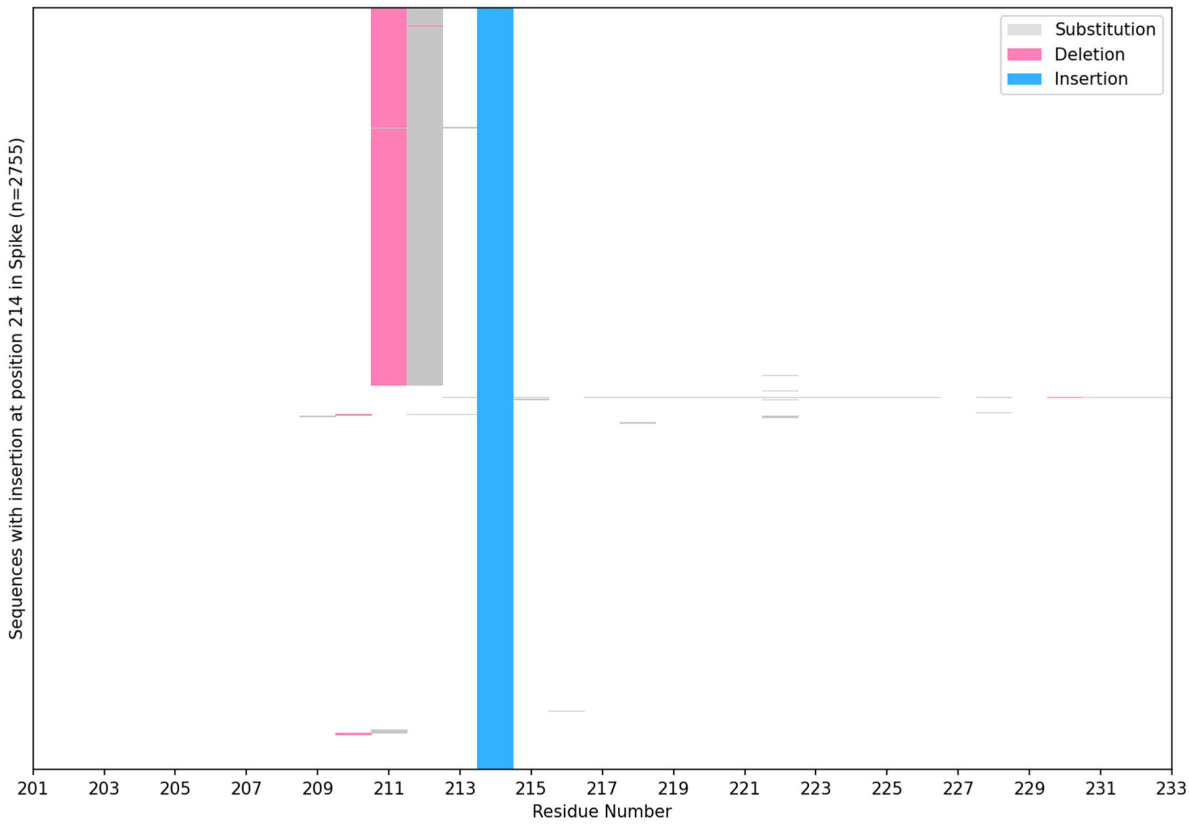

Figure S6. Occurrence of deletions and substitutions in the residues neighboring position 214, in SARS-CoV-2 genomes harboring any insertion at this position. Among all 2755 SARS-CoV-2 sequences in GISAID with any insertion at position 214 (as of December 14, 2021), we assessed the neighboring residues for the occurrence of deletions, substitutions, or other insertions. The sequences with a deletion observed at position 1358 all correspond to the Omicron variant (two sequences lack assigned lineages and one is assigned to a parent lineage), whereas other sequences containing alternate insertions at position 214 (i.e. not ins214EPE) generally have no alterations in the neighboring regions.

Table S1. Comparison of the mutations between the Omicron variant and previously identified variants of concern (VOCs) and variants of interest (VOIs). Core mutations for non-Omicron lineages were derived from the Coronavirus Antiviral Research Database (CoV-RDB). There are 26 mutations in Omicron which are not considered core mutations for any previously identify variants of concern. Among these, two were present in variants of interest (A67V and T95I) and 23 were present in another SARS-CoV-2 lineage that was not classified as a VOC or VOI (not shown in this table). Only ins214EPE was never observed in any SARS-CoV-2 sequences deposited in GISAID prior to the identification of Omicron. The first column denotes the protein domain where the mutation is present. NTD: N-terminal domain; RBD: receptor binding domain; RBM: receptor binding motif; PBCS: poly-basic cleavage site; CTD: C-terminal domain.

Domains	Position(s)	Surge Associated?	Omicron (B.1.1.529)	Alpha (B.1.1.7)	Beta (B.1.351)	Gamma (P.1)	Delta (B.1.617.2)	Lambda (C.37)	Mu (B.1.621)	Eta (B.1.525)	Iota (B.1.526)	Kappa (B.1.617.1)
NTD	5	Yes									L5F	
	18	Yes				L18F						
	19	Yes					T19R					
	20	Yes				T20N						
	26	Yes				P26S						
	52	No								Q52R		
	67	Yes	A67V							A67V		
	69	Yes	H69del	H69del						H69del		
	70	Yes	V70del	V70del						V70del		
	75	No						G75V				
	76	No						T76I				
	80	Yes			D80A							
	95	Yes	T95I						T95I		T95I	
	138	Yes				D138Y						
	142	Yes	G142D				G142D					
	143	Yes	V143del									
	144	Yes	Y144del	Y144del					Y144S	Y144del		
	145	Yes	Y145del	Y145del					Y145N	Y145del		
	156	Yes					E156G					
	157	Yes					F157del					
	158	Yes					R158del					
	190	Yes				R190S						
	211	No	N211del									
	212	No	L212I									
	**214**	**No**	**R214ins EPE**									
	215	Yes			D215G							
	241	No			L241del							
	242	Yes			L242del							
	243	Yes			A243del							
	246	Yes						R246N				
	247	Yes						S247del				

	248	No						Y248del				
	249	No						L249del				
	250	No						T250del				
	251	No						P251del				
	252	No						G252del				
	253	No						D253del			D253G	
	339	No	G339D									
	346	Yes							R346K			
	371	No	S371L									
**RBD**	373	No	S373P									
	375	No	S375F									
	417	Yes	K417N		K417N	K417T						
	440	No	N440K									
	446	No	G446S									
	452	Yes					L452R	L452Q			L452R	
	477	Yes	S477N									
	478	Yes	T478K				T478K					
	484	Yes	E484A		E484K	E484K			E484K	E484K	E484K	E484Q
**RBD (RBM)**	490	No						F490S				
	493	No	Q493K									
	496	No	G496S									
	498	No	Q498R									
	501	Yes	N501Y	N501Y	N501Y	N501Y			N501Y			
	505	No	Y505H									
	677	No								Q677H		
**PBCS**	679	No	N679K									
	681	Yes	P681H	P681H			P681R		P681H		P681R	
	547	No	T547K									
**SD1/SD2**	570	Yes		A570D								
	614	Yes	D614G	D614G	D614G	D614G	D614G	D614G	D614G	D614G	D614G	D614G
	655	Yes	H655Y			H655Y						
**Others (CTD)**	701	Yes			A701V						A701V	
	716	Yes		T716I								
	764	No	N764K									
	796	No	D796Y									
	856	No	N856K									

	859	Yes						T859N				
	888	No								F888L		
	950	Yes					D950N		D950N			
	954	No	Q954H									
	969	No	N969K									
	981	No	L981F									
	982	Yes		S982A								
	1027	Yes				T1027I						
	1071	No										Q1071H
	1118	Yes		D1118H								
	1176	Yes				V1176F						

Table S2. Number of PANGO lineages with mutations in the Omicron variant's Spike protein. The Omicron sequences deposited on November 24, 2021 that were assigned B.1 are not being considered.

AA Substitutions	Number of Pango lineage in which mutation has observed As of 14 Dec 2021
Spike_A67V	269
Spike_H69del	374
Spike_V70del	374
Spike_T95I	505
Spike_G142D	256
Spike_V143del	298
Spike_Y144del	578
Spike_Y145del	155
Spike_N211del	90
Spike_L212I	72
**Spike_ins214EPE**	**0**
Spike_G339D	66
Spike_S371L	3
Spike_S373P	64
Spike_S375F	48
Spike_K417N	161
Spike_N440K	92
Spike_G446S	94

Spike_S477N	264
Spike_T478K	328
Spike_E484A	101
Spike_Q493K	31
Spike_G496S	21
Spike_Q498R	38
Spike_N501Y	338
Spike_Y505H	16
Spike_T547K	83
Spike_D614G	1465
Spike_H655Y	372
Spike_N679K	116
Spike_P681H	376
Spike_N764K	32
Spike_D796Y	198
Spike_N856K	19
Spike_Q954H	16
Spike_N969K	26
Spike_L981F	15

Table S3. List of other insertions near amino acid position 214 of the SARS-CoV-2 Spike glycoprotein. SARS-CoV-2 genome sequences and lineage assignments were obtained from GISAID. Insertions occurring between amino acid positions 210 and 218 of the Spike glycoprotein are shown. The table is sorted first by position (first column) in ascending order and then by the genome count (last column) in descending order.

Spike Protein Amino Acid Position	Insertion	Lineage	Number of Genomes in GISAID with Insertion
210	ins210V	A.2.5.1	1
212	ins212YL	AY.119	1
213	ins213KAR	B.1.177	1
	ins213QKRL	B.1.1.271	1
	ins213SER	AY.44	1
214	ins214TDR	B.1.214.2	489
	ins214QAS	B.1.639	26
	ins214ANRN	P.1	11
	ins214VPWI	AY.4	9
	ins214TDR	B.1.214	6
	ins214HSG	AY.4	5
	ins214AAG	A.2.5	4
	ins214RRAI	B.1.1	4
	ins214KFH	B.1.1.7	3
	ins214KRI	B	3
	ins214NFG	AY.25	3
	ins214QID	B.1.214.2	3
	ins214AAG	A.2.5.2	2
	ins214AQER	B.1.429	2
	ins214RLR	AY.3	2
	ins214AAG	A.2.5.1	1
	ins214AAGGW	B.1.617.2	1
	ins214AAVE	AY.4	1
	ins214AKKN	B	1
	ins214APR	B.1.1.7	1
	ins214EAR	B.1.617.2	1
	ins214ELCD	AY.124	1
	ins214GCE	B.1.625	1
	ins214GLGG	B.1.595	1
	ins214GLKG	AY.101	1
	ins214IDR	B.1.214.2	1
	ins214KGE	B.1.1.519	1
	ins214KLGP	B.1.177	1
	ins214KRSD	B.1.1.519	1
	ins214NES	AY.51	1
	ins214NFGGG	AY.112	1
	ins214NVAHW	B.1	1

	ins214RGG	AY.100	1
	ins214SGG	B.1	1
	ins214SRA	AY.4.2.1	1
	ins214TDR	B.1.258	1
	ins214TREA	B.1.617.2	1
	ins214VRR	AY.113	1
215	ins215AGY	A.2.5	1214
	ins215AGY	A.2.5.2	389
	ins215AGY	A.2.5.1	177
	ins215AGY	A.2.5.3	32
	ins215GGD	AY.25.1	4
	ins215ADY	A.2.5	3
	ins215AGAAGY	A.2.5	1
	ins215AGY	B.1	1
	ins215ARN	AY.4	1
	ins215DRDR	B.1.214.2	1
	ins215GRL	A.2.5.3	1
	ins215KGD	AY.4.2	1
	ins215KLRS	A.2.5	1
	ins215QAFD	AY.47	1
	ins215QMR	B.1.1.7	1
	ins215RSN	B.1	1
	ins215SGY	A.2.5	1
	ins215TGY	A.2.5	1
	ins215VGY	A.2.5	1
	ins215VGY	A.2.5.2	1
	ins215Y	A.2.5	1
216	ins216ADL	B.1.2	13
218	ins218VF	B.1.2	2
	ins218VF	B.1.240	1

Table S4. Prevalence of ins214EPE in Omicron sublineages. Data corresponds to genomes deposited in GISAID and assigned to an Omicron lineage as of July 18, 2022.

Omicron Sublineage	Total Number of Genomes	Number of Genomes Containing ins214EPE	Percent of Genomes Containing ins214EPE
BA.1	2,379,108	1,841,765	77.4%
BA.2	1,925,768	193	0.01%
BA.3	1,146	5	0.4%
BA.4	49,812	1	0.0%
BA.5	139,679	0	0.0%
B.1.1.529 (not assigned to a sublineage)	7,355	638	8.7%

Table S5. SARS-CoV-2 insertions identified and attributed to template switching in the previous analysis by Garushyants, et al. All columns except for the last column are a subset of Supplementary Data 4 from the prior analysis.[14] With this information, we searched GISAID to identify the specific SARS-CoV-2 genomes in which the insertion and origin sequences were present at the specified locations. These genomes are identified in the last column. These events were considered as "positive controls" to evaluate whether homology in the preceding and/or subsequent nucleotide sequences are required for the generation of template switch-mediated insertions in SARS-CoV-2.

Insertion Sequence (5′ to 3′ on positive-sense strand)	Origin Sequence (5′ to 3′ on positive-sense strand)	Insertion Size (Nucleotides)	Insertion Genomic Position	Origin Genomic Position	Origin on Forward or Reverse Strand	Pango Lineage(s)	GISAID Sequence IDs Matching Description of Insertion from Garushyants et al.
aatgtcgcgcattgg	AATGTCGCGCATTGG	15	27631	29216	Forward	B.1.408	EPI_ISL_731651
tctgttttgctt	TCTGTTTTGCTT	12	9350	12006	Forward	B.1.2	EPI_ISL_1027726
							EPI_ISL_1028611
							EPI_ISL_1077450
							EPI_ISL_2191470
							EPI_ISL_2201947
agaaatgcctgt	AGAAATGCCCGT	12	29442	20018	Forward	B.1.1.519	EPI_ISL_2184872
						B.1.1	EPI_ISL_2184874
						B.1.1.519	EPI_ISL_2615927
ggagttcatgct	GGAGTTCATGCT	12	6638	10562	Forward	B.1.1.7	EPI_ISL_1924455
ctaaattgggta	CTAAATTGGGTA	12	28002	28166	Forward	B.1; B.1.1.294	EPI_ISL_1149442

Table S6. Sequences in the Omicron genome with a single nucleotide mismatch compared to the three candidate insertion sequences. The reference Omicron genome (EPI_ISL_6640916)[39] was obtained from GISAID. Within this genome, we identified all 9-nucleotide sequences that had only one mismatch compared to the three candidate insertion sequences (5′-GAGCCAGAA-3′; 5′-AGCCAGAAG-3′; 5′-GCCAGAAGA-3′). Each hit is shown along with the gene in which it occurs, the genome strand on which it occurs (positive sense genome or negative sense anti-genome), and the within-gene start and end genomic positions. All genomic positions are shown with respect to the positive sense genome.

Candidate Insertion Sequence (5'->3')	Sequence in Original Omicron Genome with 1 nt Mismatch (5'->3')	SARS-CoV-2 Gene	Genome Strand	Start Position	End Position
GAGCCAGAA	GAGCCTGAA	NS3	+	715	723
GAGCCAGAA	GAGCAAGAA	NSP12	-	1909	1917
GAGCCAGAA	GAACCAGAA	NSP13	+	1252	1260
GAGCCAGAA	GAGCAAGAA	NSP3	+	466	474
GAGCCAGAA	GGGCCAGAA	Nucleocapsid	+	337	345
GAGCCAGAA	GAGACAGAA	Nucleocapsid	+	1143	1151
GCCAGAAGA	ACCAGAAGA	Envelope (E)	-	216	224
GCCAGAAGA	GCCAGAGGA	Membrane (M)	-	158	166
GCCAGAAGA	GCCTGAAGA	NS3	+	717	725
GCCAGAAGA	TCCAGAAGA	NS7a	-	106	114
GCCAGAAGA	GCCACAAGA	NSP13	+	723	731
GCCAGAAGA	GCAAGAAGA	NSP3	+	468	476
GCCAGAAGA	GCCAGAAGC	Nucleocapsid	+	339	347
GCCAGAAGA	GACAGAAGA	Nucleocapsid	+	1145	1153
GCCAGAAGA	GCAAGAAGA	Spike glycoprotein (S)	-	3173	3181
AGCCAGAAG	AGCCAGAGG	Membrane (M)	-	159	167
AGCCAGAAG	AGCCTGAAG	NS3	+	716	724
AGCCAGAAG	AGCAAGAAG	NS3	-	154	162
AGCCAGAAG	AGCCACAAG	NSP1	-	149	157
AGCCAGAAG	AGCAAGAAG	NSP3	+	467	475
AGCCAGAAG	AGCCAAAAG	NSP3	-	4502	4510
AGCCAGAAG	AGCCACAAG	NSP5	-	694	702

AGCCAGAAG	GGCCAGAAG	Nucleocapsid	+	338	346
AGCCAGAAG	AGACAGAAG	Nucleocapsid	+	1144	1152
AGCCAGAAG	AACCAGAAG	Spike glycoprotein (S)	-	2639	2647

Table S7. Number of genomes with exact matches to candidate insertions by Pango lineage. For all three possible 9-nucleotide sequences corresponding to ins214EPE (5′-GAGCCAGAA-3′; 5′-AGCCAGAAG-3′; 5′-GCCAGAAGA-3′), we searched for SARS-CoV-2 genomes in GISAID with exact matches in the genome (positive sense) or anti-genome (negative sense). The number of such matches is shown for each Pango lineage with at least one match. Because Omicron corresponds to the Pango lineage B.1.1.529, other descendants of B.1.1 are shown in red, and B.1.1 itself is shown in red, italics, and bold.

5'-GAGCCAGAA-3'				5'-AGCCAGAAG-3'				5'-GCCAGAAGA-3'			
Positive (+) sense	No. of genomes	Negative (-) sense	No. of genomes	Positive (+) sense	No. of genomes	Negative (-) sense	No. of genomes	Positive (+) sense	No. of genomes	Negative (-) sense	No. of genomes
BA.1	1169	AY.44	13	BA.1	1169	B.1.1.7	33	BA.1	1169	AY.25	196771
AY.47	227	AY.25	6	B.1.177.60	27	***B.1.1***	***27***	B.1.1.7	28	Unassigned	1523
AY.4.2.1	117	B.1.177	2	B.1.1.7	16	AY.103	20	B.1.177.60	27	AY.39	352
B.1.1.176	81	B.1.1.216	1	AY.45	13	AY.122	16	AY.4	15	AY.113	296
AY.122	48	B.1.582	1	B.1.617.2	8	B.1	13	B.1.1.397	13	B.1.617.2	269
AY.4	39	AY.13	1	AY.4	6	AY.4	11	AY.45	13	AY.122	258
B.1.1.7	35	AY.4	1	AY.103	5	AY.20	10	B.1.617.2	9	AY.4	215
P.1.15	30	AY.122	1	Unassigned	4	P.1	10	Unassigned	6	AY.25.1	192
AY.29	30	B.1.2	1	P.1	4	B.1.427	9	AY.44	4	AY.43	166
B.1.177.60	27			AY.125	3	B.1.2	9	P.1	4	AY.44	147
B.1.2	24			AY.43	3	AY.25	9	AY.125	3	AY.75	110
B.1	23			AY.44	2	AY.39	7	AY.43	3	AY.46.6	100
B.1.1.519	18			B.1.177.21	1	AY.3.1	7	AY.3	2	B.1.1.7	87
AY.16	18			B.1.1.39	1	AY.44	6	AY.36	2	AY.120	85
AY.45	17			B.1.320	1	AY.43	6	AY.4.2	2	AY.103	78
B.1.617.2	16			B.40	1	AY.3	5	AY.46.5	1	B	66
B.1.1	***14***			B.1.36.8	1	AY.26	5	AY.118	1	AY.42	65
AY.103	13			AY.4.1	1	A.21	5	AY.39.1	1	AY.114	63
AY.43	12			AY.46.5	1	Unassigned	4	AY.4.2.1	1	AY.33	55
AY.46	11			B	1	B.1.617.2	4	B.1	1	AY.121	47
AY.9	10			AY.4.2	1	AY.9.2	3	AY.25	1	AY.92	43
Unassigned	8			AY.36	1	B.1.1.519	3	AY.33	1	AY.126	41
P.1	7			***B.1.1***	***1***	B.1.258.17	3	AY.122	1	AY.98.1	38

AY.44	7			A.2.5	1	AY.45	2	AY.126	1	AY.45	33
AY.14	5			B.1	1	B.1.177.21	2	AY.29	1	AY.3	29
AY.25	5			AY.4.2.1	1	AY.114	2	AY.4.1	1	B.1	27
AY.113	4			AY.39.1	1	AY.125	2	A.2.5	1	AY.5.4	25
AY.75	3					B.1.609	2	***B.1.1***	***1***	AY.101	24
AY.91	3					AY.61	2	B.1.177.77	1	AY.20	19
AY.88	3					AY.27	2	B.1.36.8	1	AY.88	18
AY.127	3					P.1.2	2	B.1.320	1	AY.99	18
B.1.177.21	3					B.1.1.306	2	B.1.1.39	1	AY.99.2	17
AY.125	3					B.1.1.214	2	B.40	1	AY.108	15
AY.3	2					B.1.621	1	AY.54	1	AY.102	14
AY.100	2					AY.77	1			AY.22	13
B.1.429	2					AY.29.1	1			AY.46.4	12
AY.121	2					AY.36	1			AY.3.1	12
AY.4.2	2					B.1.617.1	1			AY.124	12
B.40	2					B.1.36	1			B.1.2	11
P.1.14	2					AY.121	1			AY.100	10
B.1.36.39	2					B.1.582	1			AY.106	10
AY.4.6	2					AY.124	1			C.37	10
B.1.1.28	2					AY.98	1			AY.26	9
B.1.621.1	2					B.1.1.161	1			AY.47	9
C.30	2					B.1.177	1			AY.43.3	9
AY.4.2.2	2					B.1.351	1			AY.82	7
AY.46.6	2					B.1.221.4	1			B.1.1	7
B.1.221	2					B.1.1.269	1			AY.93	7
AY.98	1					B.1.526	1			B.1.429	7
AY.46.5	1					AY.13	1			AY.9.2	7
AY.36	1					B.1.1.271	1			AY.85	6
AY.39	1					B.1.1.449	1			AY.43.1	6
AY.13	1					B.1.596	1			AY.119	5
AY.122.1	1					B.1.637	1			AY.70	5

B.1.177.81	1					B.1.320	1			AY.95	5
B.1.351	1					B.40	1			B.1.582	5
AY.39.1	1					AY.39.1.1	1			AY.96	4
AY.126	1									AY.5.3	4
AY.4.1	1									AY.34.1	4
AY.5.2	1									AY.4.5	4
AY.57	1									B.1.637	4
AY.124	1									AY.39.1	4
B.1.36.8	1									AY.120.1	3
B.1.320	1									AY.46	3
B.1.1.39	1									AY.1	3
B.1.426	1									AY.73	3
B.1.147	1									AY.57	3
B.1.258	1									AY.109	3
AY.61	1									AY.30	3
B.1.575	1									B.1.177	2
B.1.1.89	1									B.1.1.214	2
AY.108	1									AY.84	2
AY.19	1									AY.6	2
AY.24	1									AY.123	2
A.2.5	1									AY.77	2
B.1.398	1									AY.43.4	2
AY.84	1									AY.23	2
B.1.561	1									B.1.565	2
AY.37	1									AY.118	2
B.1.36	1									AY.125	2
AY.98.1	1									AY.19	2
C.37	1									AY.4.2.3	2
B	1									AY.5	2
AY.20	1									AY.110	2
AY.34	1									AY.78	2

										B.1.1.526	2
										P.1	2
										B.1.177.21	2
										B.1.160.22	2
										AY.79	2
										B.1.596	2
										AY.54	2
										AY.111	2
										AY.59	2
										AY.61	1
										B.55	1
										AY.34.2	1
										AY.49	1
										B.1.415.1	1
										B.1.619	1
										AY.24	1
										AY.14	1
										B.1.254	1
										AY.53	1
										B.1.581	1
										B.1.459	1
										AY.98	1
										AY.4.2	1
										AY.67	1
										AY.4.2.1	1
										AY.18	1
										AY.32	1
										AY.116	1
										B.1.320	1
										AY.117	1
										AY.7.2	1

										AY.13	1
										C.37.1	1
										AY.58	1
										AY.127	1
										B.1.466.2	1
										AY.90	1
										B.1.535	1
										AY.76	1
										P.2	1
										AY.46.2	1
										B.1.1.519	1
										A.2.5	1
										B.1.177.81	1
										B.1.36.38	1
										P.1.1	1
										B.1.469	1
										AY.27	1
										B.1.1.449	1
										B.1.1.153	1
										AY.69	1
										AY.50	1
										AY.29	1
										B.1.1.271	1
										B.1.621	1
										AY.39.1.1	1

Table S8. Number of seasonal or enteric *Coronaviridae* genomes with exact matches to each candidate insertion sequence. We searched non-SARS-CoV-2 *Coronaviridae* genomes and anti-genomes for exact matches to all three possible 9-nucleotide sequences (5′-GAGCCAGAA-3′; 5′-AGCCAGAAG-3′; 5′-GCCAGAAGA-3′) that gave rise to ins214EPE in Omicron. This table shows the number of matches that were identified for each such virus-gene combination with at least one match, along with the strand (positive sense genome or negative sense anti-genome) on which the match was found.

Candidate Insertion Sequence (5'->3')	Genome Strand	Virus	Gene	Number of Genomes with Match
GCCAGAAGA	+	Human coronavirus OC43	Nucleocapsid (N)	177
GCCAGAAGA	+	Human enteric coronavirus strain 4408	Nucleocapsid (N)	2
GCCAGAAGA	+	Human coronavirus OC43	Protein I (internal to N)	6
GAGCCAGAA	+	Human coronavirus OC43	Replicase polyprotein 1a (ORF1a)	98
GAGCCAGAA	+	Human enteric coronavirus strain 4408	Replicase polyprotein 1a (ORF1a)	1
GAGCCAGAA	-	Human coronavirus 229E	Spike glycoprotein (S)	33
GCCAGAAGA	-	Human coronavirus NL63	Spike glycoprotein (S)	2

Table S9. Coexpression analysis of ACE2 and ANPEP in single cell RNA-sequencing datasets. The number and percent of cells in each category expressing ACE2, ANPEP, or both ACE2 and ANPEP are shown, along with the observed to expected ratio of co-expression for the given category. In the first row, "All studies" corresponds to the set of studies that are hosted in the previously described Single Cell application at academia.nferx.com.[46,76] The analyzed enterocytes are derived from a study of ileal biopsies from Crohn's Disease patients.[78] The analyzed respiratory ciliated cells and FOXN4+ respiratory epithelial cells are derived from a study of nasopharyngeal and bronchial samples from COVID-19 patients and healthy controls.[77]

Study source (PMID)	Cell Type	Number of cells	Cells expressing ACE2 (%)	Cells expressing ANPEP (%)	Cells co-expressing ACE2 and ANPEP (%)	Observed / Expected Ratio
All studies	*All cells*	*2.8M*	*26.7K (0.95%)*	*213.1K (7.6%)*	*13.5K (0.48%)*	*6.6*
Crohn's Disease Small Intestine (PMID 31474370)	*All cells*	*32.5K*	*432 (1.3%)*	*1.4K (4.3%)*	*403 (1.2%)*	*21.7*
	Enterocytes	809	337 (41.7%)	757 (93.6%)	329 (40.7%)	1.04
COVID-19 Respiratory Tract (PMID 32591762)	*All cells*	*135.6K*	*3K (2.2%)*	*15.4K (11.4%)*	*1.5K (1.1%)*	*4.4*
	Respiratory ciliated cells	5.8K	827 (14.3%)	1.2K (20.7%)	217 (3.7%)	1.3
	FOXN4+ respiratory epithelial cells	787	89 (11.3%)	302 (38.4%)	56 (7.1%)	1.6

Table S10. Coexpression analysis of ACE2 and DPP4 in single cell RNA-sequencing datasets. The number and percent of cells in each category expressing ACE2, DPP4, or both ACE2 and DPP4 are shown, along with the observed to expected ratio of co-expression for the given category. In the first row, "All studies" corresponds to the set of studies that are hosted in the previously described Single Cell application at academia.nferx.com.[46,76] The analyzed enterocytes are derived from a study of ileal biopsies from Crohn's Disease patients.[78] The analyzed respiratory ciliated cells and FOXN4+ respiratory epithelial cells are derived from a study of nasopharyngeal and bronchial samples from COVID-19 patients and healthy controls.[77]

Study source (PMID)	Cell Type	Number of cells	Cells expressing ACE2 (%)	Cells expressing DPP4 (%)	Cells co-expressing ACE2 and DPP4 (%)	Observed / Expected Ratio
All studies	*All cells*	*2.8M*	*26.7K (0.95%)*	*112.5K (4.02%)*	*6.4K (0.23%)*	*6.0*
Crohn's Disease Small Intestine (PMID 31474370)	*All cells*	*32.5K*	*432 (1.3%)*	*1.3K (4.9%)*	*175 (0.54%)*	*10.1*
	Enterocytes	809	337 (41.7%)	254 (31.4%)	153 (18.9%)	1.4

Table S11. Local alignment and homology scores for nucleotides upstream or downstream of origin and insertion sites from previously identified template switch-mediated insertions. These correspond to five "positive control" SARS-CoV-2 template switch-mediated insertions shown in Table S6, which were identified in the prior analysis by Garushyants, et al.[14] We identified the SARS-CoV-2 genomes in GISAID corresponding to each insertion, and then calculated normalized homology scores (NHS) for the 35 or 7 nucleotides upstream or downstream from each insertion and origin sequence. The scores along with the alignments are shown here, and the scores are compared to a null distribution of NHS values computed between 10,000 pairs of randomly selected non-overlapping 35- or 7-nucleotide sequences from the reference SARS-CoV-2 genome in **Figure S3**.

<table>
<tr><th>Insertion Sequence (5′ to 3′)</th><th>Sequence IDs</th><th>Upstream Normalized Homology Score
(35 nt Score; 7-nt Score)</th><th>Downstream Normalized Homology Score
(35-nt Score; 7-nt Score)</th><th>Upstream Alignment (5′to 3′)</th><th>Downstream Alignment (5′ to 3′)</th></tr>
<tr><td>AATGTCGCGCATTGG</td><td>EPI_ISL_731651</td><td>57.14;
42.86</td><td>60;
42.86</td><td>TGACGGCGTAA-AA----C----A-CGTCTATCAGTTACGTG-C--CA--
|| ||| | ||||||||| ||| | |
-----GC---ACAATTTGCCCCCAGCG-CT-TCAG---CGT-TCTTC-GG</td><td>AAC-TGTTC-ATCAGA-CA-A-----GAGGAA-GT--TCAA-GAACT-T
||| | || ||| |||||| | | ||| |
--CATG---GA--AG-TCACACCTTCG-GGAACGTGGT---TG-AC-CT</td></tr>
<tr><td rowspan="5">TCTGTTTTGCTT</td><td>EPI_ISL_1027726</td><td>62.86;
71.43</td><td>62.86;
57.14</td><td>TTTCTGTGG--TGTAGATGC-TGT--AAA---T--TT--ACTTACTA-
| | |||||||| ||| | || ||||||
---C--T--ACTG-A-A-GCCT-TTGAAAAAATGGTTTCAC-TACT-T</td><td>AT--ATG-----T--T-TAC-ACCACTAATT-CAAC-CTATTGGTGCT
| ||| | | || |||||| ||| | || ||
-TCCATGCAGGGTGCTGTA-GA-CA-TAA--ACAA-GC--TT--TG--</td></tr>
<tr><td>EPI_ISL_1028611</td><td>62.86;
71.43</td><td>62.86;
57.14</td><td>TTTCTGTGG--TGTAGATGC-TGT--AAA---T--TT--ACTTACTA-
| | |||||||| ||| | || ||||||
---C--T--ACTG-A-A-GCCT-TTGAAAAAATGGTTTCAC-TACT-T</td><td>AT--ATG-----T--T-TAC-ACCACTAATT-CAAC-CTATTGGTGCT
| ||| | | || |||||| ||| | || ||
-TCCATGCAGGGTGCTGTA-GA-CA-TAA--ACAA-GC--TT--TG--</td></tr>
<tr><td>EPI_ISL_1077450</td><td>62.86;
71.43</td><td>62.86;
57.14</td><td>TTTCTGTGG--TGTAGATGC-TGT--AAA---T--TT--ACTTACTA-
| | |||||||| ||| | || ||||||
---C--T--ACTG-A-A-GCCT-TTGAAAAAATGGTTTCAC-TACT-T</td><td>AT--ATG-----T--T-TAC-ACCACTAATT-CAAC-CTATTGGTGCT
| ||| | | || |||||| ||| | || ||
-TCCATGCAGGGTGCTGTA-GA-CA-TAA--ACAA-GC--TT--TG--</td></tr>
<tr><td>EPI_ISL_2191470</td><td>62.86;
71.43</td><td>62.86;
57.14</td><td>TTTCTGTGG--TGTAGATGC-TGT--AAA---T--TT--ACTTACTA-
| | |||||||| ||| | || ||||||
---C--T--ACTG-A-A-GCCT-TTGAAAAAATGGTTTCAC-TACT-T</td><td>AT--ATG-----T--T-TAC-ACCACTAATT-CAAC-CTATTGGTGCT
| ||| | | || |||||| ||| | || ||
-TCCATGCAGGGTGCTGTA-GA-CA-TAA--ACAA-GC--TT--TG--</td></tr>
<tr><td>EPI_ISL_2201947</td><td>62.86;
71.43</td><td>62.86;
57.14</td><td>TTTCTGTGG--TGTAGATGC-TGT--AAA---T--TT--ACTTACTA-
| | |||||||| ||| | || ||||||
---C--T--ACTG-A-A-GCCT-TTGAAAAAATGGTTTCAC-TACT-T</td><td>AT--ATG-----T--T-TAC-ACCACTAATT-CAAC-CTATTGGTGCT
| ||| | | || |||||| ||| | || ||
-TCCATGCAGGGTGCTGTA-GA-CA-TAA--ACAA-GC--TT--TG--</td></tr>
<tr><td rowspan="3">AGAAATGCCTGT</td><td>EPI_ISL_2184872</td><td>54.29;
28.57</td><td>62.86;
85.71</td><td>---ACTC---A-AGCCTTACCGCA-GAGA-CAGAAGAAAC-AG-C--A---
|| ||| || |||| | ||| ||| |
TTGA-T-GGTAGAG--TT---G-ATG-G-TC--AAG----TAGACTTATTT</td><td>AACTGTGACTC-TTCTTCC-TGCTG-CAGATTT-GG-A-TG-------
|| || | | ||||| | | |||| || | ||
AA-TG-G--T-GTTCTT--AT--T-ACAGA---AGGTAGTGTTAAAGG</td></tr>
<tr><td>EPI_ISL_2184874</td><td>54.29;
28.57</td><td>62.86;
85.71</td><td>---ACTC---A-AGCCTTACCGCA-GAGA-CAGAAGAAAC-AG-C--A---
|| ||| || |||| | ||| ||| |
TTGA-T-GGTAGAG--TT---G-ATG-G-TC--AAG----TAGACTTATTT</td><td>AACTGTGACTC-TTCTTCC-TGCTG-CAGATTT-GG-A-TG-------
|| || | | ||||| | | |||| || | ||
AA-TG-G--T-GTTCTT--AT--T-ACAGA---AGGTAGTGTTAAAGG</td></tr>
<tr><td>EPI_ISL_2615927</td><td>54.29;
28.57</td><td>62.86;
85.71</td><td>---ACTC---A-AGCCTTACCGCA-GAGA-CAGAAGAAAC-AG-C--A---
|| ||| || |||| | ||| ||| |
TTGA-T-GGTAGAG--TT---G-ATG-G-TC--AAG----TAGACTTATTT</td><td>AACTGTGACTC-TTCTTCC-TGCTG-CAGATTT-GG-A-TG-------
|| || | | ||||| | | |||| || | ||
AA-TG-G--T-GTTCTT--AT--T-ACAGA---AGGTAGTGTTAAAGG</td></tr>
<tr><td>GGAGTTCATGCT</td><td>EPI_ISL_1924455</td><td>57.14;
57.14</td><td>60;
28.57</td><td>ATCTAGAGTATTAGGT-TTGAAA-A--C-CC-T-TGC---T-ACTC----
|| ||| ||| || |||||| |||| |
--CT----T-TT---TGTT---ACATGCACCATATG-GAATTAC-CAACT</td><td>ATGGTTT-AGCT-G-CTGTTA-ATAGTGTCC--CTTG----GGA--T--
|| || || ||||||||| ||| ||| |
--GG---CA-C-AGAC--TTAGA-AG-GT--AACTT-TTATGGACCTTT</td></tr>
</table>

CTAAATTG GGTA	EPI_ISL_1149 442	N/A	N/A	Region had low quality sequence	Region had low quality sequence
